# Indications for Trephining

**Published:** 1891-08

**Authors:** 


INDICATIONS FOR TREPHINING.—John B. Deaver, in the *Annals of Surgery*, gives the following indications for trephining:

1. Simple depressed fracture with or without brain symptoms.
2. Compound depressed fracture with or without brain symptoms.
3. Impacted fracture, simple or compound, with or without brain symptoms.
4. Comminuted fracture, simple or compound, with or without brain symptoms.
5. Compound fissured fracture with depression of bone without brain symptoms.
6. Compound fissured fracture with depression of bone with brain symptoms.
7. Compound fissured fracture without depression of bone and without brain symptoms, in which there is blending through the fissure or fissures.
8. All punctured, incised and gunshot fractures.—*Practice.*

